# Diphtheria Outbreaks in Schools in Central Highland Districts, Vietnam, 2015–2018 

**DOI:** 10.3201/eid2603.191027

**Published:** 2020-03

**Authors:** Noriko Kitamura, Thao T.T. Le, Lien T. Le, Luong D. Nguyen, Anh T. Dao, Thanh T. Hoang, Keisuke Yoshihara, Makiko Iijima, Tran M. The, Hung M. Do, Huy X. Le, Hung T. Do, Anh D. Dang, Mai Q. Vien, Lay-Myint Yoshida

**Affiliations:** Nagasaki University, Nagasaki, Japan (N. Kitamura, K. Yoshihara, L.-M. Yoshida);; Pasteur Institute in Nha Trang, Nha Trang, Vietnam (T.T.T. Le, L.T. Le, L.D. Nguyen, A.T. Dao, T.T. Hoang, T.M. The, H.M. Do, H.X. Le, H.T. Do, M.Q. Vien);; World Health Organization Representative Office for Vietnam, EPI, Hanoi, Vietnam (M. Iijima);; National Institute of Hygiene and Epidemiology, Hanoi (A.D. Dang)

**Keywords:** Corynebacterium diphtheriae, bacteria, diphtheria, outbreaks, epidemic, epidemiology, multilocus sequence typing, schools, central highland districts, Vietnam

## Abstract

During 2015–2018, seven schools in rural Vietnam experienced diphtheria outbreaks. Multilocus sequence types were the same within schools but differed between schools. Low vaccine coverage and crowded dormitories might have contributed to the outbreaks. Authorities should consider administering routine vaccinations and booster doses for students entering the school system.

Diphtheria is a serious childhood disease with a high mortality rate (*1*). After a diphtheria–tetanus–pertussis vaccine (DTP) was introduced in the early 20th century, the number of cases dramatically decreased. Incidence reached a low of 4,333 cases in 2006, but more recently, the number of reported cases has increased, with incidence reaching 16,648 cases in 2018 (*2*). 

In 1981, Vietnam introduced a vaccination program in which participants received 3 primary doses of DTP (DTP3) vaccine; in 2011, a booster shot (DTP4) to be given 18 months after the initial doses was added (*3*). Although diphtheria cases had become sporadic by 2010, beginning in 2013, outbreaks occurred in the western and central highland areas of Vietnam, which prompted our study (*4*). 

## The Study

**Figure 1:**
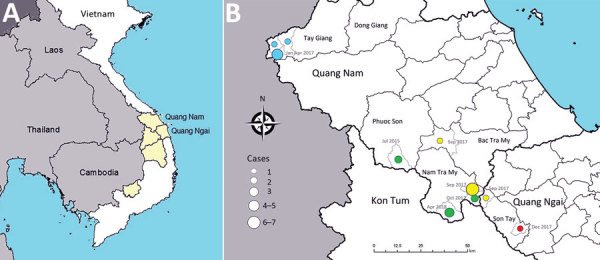
A) Provinces where diphtheria cases were identified in Vietnam in 2010s. Diphtheria cases were reported from provinces (shaded) neighboring Laos or Cambodia. B) Laboratory-confirmed diphtheria cases in the central highland region of Quang Nam Province and Quang Ngai Province, central Vietnam, 2015–2018. Colored circles indicate separate outbreaks. Source: https://gadm.org/download_country_v3.html

During June 2015–April 2018, the Pasteur Institute in Nha Trang, Vietnam, and the provincial health authority investigated 46 cases involving patients with suspected diphtheria, 8 of whom died, and 49 asymptomatic contacts in the provinces of Quang Nam and Quang Ngai in the central highlands region of Vietnam (Figure 1). We used standard case investigation forms to collect demographic and clinical information. We collected throat swab specimens from 93 patients and contacts but were unable to collect samples from 2 patients who had died. No cutaneous diphtheria was reported.

We used sheep blood agar and tellurite medium cultures to identify *Corynebacterium diphtheriae* and extracted DNA with a QIAGEN DNA Mini Kit (QIAGEN, https://www.qiagen.com), following a standard protocol. We used 2 sets of primers, Tox1/Tox2 and Dipht6F/Dipht6R, for PCR testing (*5*). The Elek test for diphtheria is not available in Vietnam.

Laboratory testing confirmed diphtheria in 22 of 46 suspected cases: 17 patients, including 4 who died, tested positive in both culture and PCR tests, whereas 5 patients, including 1 who died, tested positive only by PCR. We categorized diagnosis as epidemiologic for 10 patients for whom PCR results were not available, 7 suspected cases and 3 in which the person died. We confirmed 2 of 49 asymptomatic contacts as carriers of diphtheria (*6*). 

We used Api Coryne (bioMérieux, https://www.biomerieux.com) to identify biotypes of *C. diphtheriae* isolates; 15 of 17 culture-positive isolates were biotype *mitis* and 1 each was *gravis* and *intermedius*. We conducted multilocus sequence typing (MLST) by using 7 primer sets for *C. diphtheriae* housekeeping genes according to reported protocol (*7*). Using the *C. diphtheriae* MLST database (https://pubmlst.org/cdiphtheriae), we detected 4 sequence types (STs): ST67 (n = 7), ST209 (n = 9), ST243 (n = 7), and ST244 (n = 1).

Among the 31 patients with confirmed or suspected diphtheria, 21 (60%) were male; age range was 1–45 years (median 10 years). We summarized case characteristics (Table 1) and epidemiologic links and STs by cluster (Table 2). The most common symptoms recorded were fever (82%), followed by pseudomembrane and difficulty swallowing (76%).

**Table 1:** Characteristics of confirmed and epidemiologically linked cases of diphtheria, central highlands of Vietnam, 2015–2018*

Characteristic	Confirmed	Epidemiologically linked	Epidemiologically linked asymptomatic carriers	Total
Age, y				
<1	0 (0)	0 (0)	0 (0)	0 (0)
1–4	2 (9)	1 (10)	1 (50)	4 (12)
5–9	7 (32)	2(20)	1 (50)	10 (29)
10–14	9 (41)	0 (0)	0 (0)	9 (26)
15–19	3 (14)	4(40)	0 (0)	7 (21)
≥20	1 (5)	3(30)	0 (0)	4 (12)
Sex				
M	14 (64)	6 (55)	1 (50)	21 (60)
F	9 (36)	4 (45)	1 (50)	14 (40)
Vaccination history, no. doses				
0	9 (41)	9 (90)	0 (0)	18 (51)
1	0 (0)	0 (0)	1 (50)	1 (3)
2	1 (5)	0 (0)	0 (0)	1 (3)
≥3	3 (14)	0 (0)	1 (50)	4 (11)
Unknown	9 (41)	1 (10)	0 (0)	11 (31)
Symptoms				
Fever	18 (81)	10 (100)	NA	28 (82)
Sore throat	15 (68)	10 (100)	NA	25 (74)
Pseudomembrane	17 (77)	9 (90)	NA	26 (76)
Difficulty swallowing	14 (64)	10 (100)	NA	26 (76)
Submandibular LN swelling	14 (64)	6 (60)	NA	20 (59)
Death	5 (23)	3 (30)	NA	8 (24)
Total no./no. persons investigated (%)	22/46 (48)	10/46 (22)	2/49 (4)	34/95 (36)

*Values are no. (%) except as indicated; total no. indicated number of patients with confirmed or suspected diphtheria or with diphtheria carrier status. LN, lymphadenopathic.

**Table 2:** Epidemiologic link and MLST results for 34 confirmed or epidemiologically linked case-patients with diphtheria, central highlands of Vietnam, 2015–2018*

District	Date of symptom onset	Patient age, y/sex	Epidemiologic link	Vaccine status†	Died	Culture result	PCR	ST	Biotype	Case
Phuoc Son	2015 Jun 30	26/F	Patient 1	UNK	X	ǂ	ǂ			Linked
	2015 Jun 30	18/M		UNK		–	§			Linked
	2015 Jul 4	17/F		UNK	X	–	§			Linked
	2015 Jul 4	27/M	Patient 1’s husband	UNK		+	+§	67	*mitis*	Confirmed
	2015 Jul 4	16/M		UNK		–	§			Linked
	2015 Jul 5	7/M	Patient 1’s son	UNK		–	§			Linked
	2015 Jul 5	20/M		UNK		–	§			Linked
	2015 Jul 8	45/M		UNK		–	§			Linked
	2015 Jul 9	1/F		UNK		–	§			Linked
	2015 Jul 14	14/M		UNK		+	+§	67	*mitis*	Confirmed
	2015 Jul 14	9/F		UNK		–	§			Linked
Tay Giang	2017 Jan 10	16/M	Tay Giang HS	UNK	X	ǂ	ǂ			Linked
	2017 Jan 10	17/M		UNK	X	+	+	243	*mitis*	Confirmed
Son Tay	2017 Mar 15	13/M		3	X	+	+	209	*mitis*	Confirmed
Tay Giang	2017 Apr 20	7/M		4	X	+	+	243	*mitis*	Confirmed
	2017 Apr 22	15/F		UNK		+	+	243	*mitis*	Confirmed
	2017 Apr 25	7/M	Gari PS	UNK		+	+	243	*mitis*	Confirmed
	2017 May 20	10/M	Patient 2 (Gari SS)	UNK		+	+	243	*mitis*	Confirmed
	2017 May 20	10/M	Gari SS	3		+	+	243	*mitis*	Confirmed
	2017 May 23	15/M	Patient 2’s brother’s friend	0		+	+	243	*mitis*	Confirmed
Bac Tra My	2017 Sep 5	5/F		UNK		–	+	209		Confirmed
Nam Tra My	2017 Sep 27	12/M	Tra Van SS	UNK		+	+	209	*mitis*	Confirmed
	2017 Sep 27	8/M	Tra Van PS	0	X	+	+	209	*mitis*	Confirmed
	2017 Sep 30	9/F		0		–	+	209		Confirmed
	2017 Sep 30	10/F		0		–	+	209		Confirmed
	2017 Sep 30	8/F		0		–	+	209		Confirmed
	2017 Oct 3	11/F		0		+	+	209	*mitis*	Confirmed
	2017 Oct 3	10/M		0		+	+	209	*mitis*	Confirmed
Nam Tra My	2017 Oct 8	12/F	Tra Vinh SS	UNK		–	+	67		Confirmed
	2017 Oct 12	13/M		UNK		+	+	67	*mitis*	Confirmed
Son Tay	2017 Dec 24	3/F		UNK		+	+	244	*gravis*	Confirmed
Nam Tra My	2018 Apr 17	4/M	Man Di SS	2	X	–	+	67	*intermed*	Confirmed
	2018 Apr 24	4/M		3 + 1 SIA		+	+	67		Linked
	2018 Apr 24	5/F		1 + 1 SIA		–	+	67		Linked

*Biotypes are of *Corynebacterium diphtheriae* bacteria. HS, high school; MLST, multilocus sequence typing; SIA, supplemental immunization activity; PS, primary school; SS, secondary school; ST, sequence type; UNK, unknown; –, negative; +, positive. †The time of last vaccination was infancy or at 1 y of age according to the vaccination program in Vietnam. SIA dose was given October 30, 2017, for the last 2 case-patients. ‡Culture and PCR were not performed for these persons because their samples were not collected. §PCR was not conducted for these 10 case-patients because this technique was not available during 2015. Stored isolates from 2 culture-positive case-patients were tested by PCR in 2017.

We determined geographic areas in which cases were identified (Figure 1). Most residents in the central highlands area were in ethnic minority groups. Healthcare access is limited because of mountainous terrain and social barriers. In this area, each commune has a primary and a secondary school, but 10 communes share 1 district-level high school. All students, from primary through high school, live in dormitories during the week, and 30–50 students might live in a ≈50 m^2^ room.

**Figure 2:**
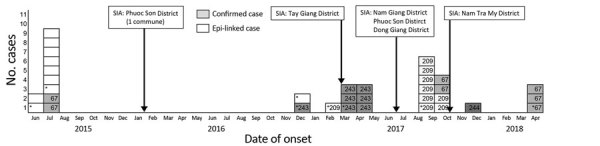
Confirmed and probable cases of diphtheria identified during June 2015–April 2018 in Vietnam. Numbers indicate multilocus sequencing type of confirmed cases with sequence types (STs) ST67, ST 243, ST209, and ST244 (gray shading). White indicates epidemiologically linked cases, and asterisks indicate cases in which the patient died. Epi, epidemiologically; SIA, supplemental immunization activity.

After January 2017, in each commune, diphtheria clusters formed mainly by school; cases in each school-based cluster shared the same ST. School clusters of the same ST in 2 communes in Tay Giang District were linked by a student who commuted between the communes. We could not identify any other epidemiologic links between clusters. An epidemic curve (Figure 2) showed the ST and outcome of cases by their onset. A long gap between clusters might indicate that the disease was transmitted through asymptomatic or skin carriers. However, further genomic testing is necessary to clarify the transmission pathway. 

Of 8 persons who died, 3 were vaccinated, 1 each with 2, 3, and 4 doses. However, the vaccination history of 85% of patients was unknown. To compensate for the lack of vaccination history, we obtained administrative details of vaccination coverage in Nam Tra My District during 2013–2016. Of the 10 communes, only 3 (Tra Van, Tra Vinh, and Tra Nam) reported cases. We compared the ratios of vaccinated and unvaccinated children and found a significantly smaller proportion of children had received DTP3 in the outbreak communes than in nonoutbreak communes (57% [95% CI 53.3%–61.2%] vs. 77% [95% CI 87.0%–90.1%]; p<0.05 by χ^2^ test).

## Conclusions

Our investigation detected 22 patients with laboratory-confirmed *C. diphtheriae* cases during 2015–2018 in this region of Vietnam, 83% of whom were >5 years of age. It has been predicted that age of diphtheria case-patients could increase after introduction of DTP because a high proportion of older persons will be susceptible to the disease due to reduced circulation of bacteria, especially when no booster dose is provided (*8*). The 4 MLST types identified in this study (ST67, ST209, ST243 and ST244) were also identified in Thailand, Cambodia, the Philippines, and Binh Phuoc Province in Vietnam in the 2010s (*4*,*9*,*10*). We found only 1 ST in each cluster location, which might indicate 1 person as the source of infection in each location. In addition, we identified no clear epidemiologic link among clusters. Detecting different STs between clusters indicates that multiple strains of *C. diphtheriae* were circulating in Vietnam, as well as in neighboring countries. This transmission pattern might not have changed since the prevaccination era when diphtheria was reported to spread from school to school or neighborhood to neighborhood (*11*).

The reemergence of diphtheria in Vietnam raises several concerns. Administrative coverage, although not always accurate, indicated DTP3 coverage of 57%, possibly creating a larger pool of susceptible people. In 2013, the health service temporarily suspended DTP immunization during a severe adverse event case investigation, which halved DTP3 coverage in the country (*2*) and potentially led to outbreaks. Students also share crowded school dormitories, which is a major factor for spreading disease. Moreover, students go home on weekends, increasing the chance of transmission between their schools and homes. Our finding of vaccinated people dying is particularly alarming because it might indicate a waning of vaccine-derived immunity.

Several interventions were conducted to control outbreaks. Erythromycin tablets were distributed to all contacts of diphtheria patients. However, only 2 asymptomatic carriers were identified among 49 contacts, lower than expected considering that 97% of case-patients could be asymptomatic in a vaccinated population (*12*). However, the sensitivity of laboratory testing might have been low because of the length of time required to collect and transport samples or because of prior antimicrobial drug use, so some carriers likely were not identified. 

Supplemental immunization activities were conducted in the outbreak area and 2 neighboring districts (Nam Giang and Dong Giang). Healthcare agencies initiated 2 campaigns: the first, targeting persons 5–40 years of age, sought to administer 3 doses of tetanus–diphtheria vaccine and achieved >90% coverage. Simultaneously, a second campaign was conducted to administer DPT to previously unvaccinated children 1–4 years of age. However, 1 unvaccinated person with diphtheria and 2 asymptomatic carriers who had received 1 dose of DPT were reported 6 months after the supplemental immunization activity. This finding was probably because diphtheria toxoid vaccine does not prevent transmission but prevents respiratory disease (*13*); thus, carriage of the organism persists.

Although Vietnam has maintained high DTP3 coverage nationally, efforts should be intensified to increase coverage in specific areas of the country (*14*). Persistent immunity resulting from DTP3 alone is not apparent (*14*), and immunity might wane before children start school (*15*). The World Health Organization recommends that students receive a booster vaccination when entering school (*15*). However, even if this recommendation is adopted, maintaining high uptake of primary and booster doses remains critical.
